# Sleep disturbance and problematic internet use in adolescents with and without major depressive disorder: a comparative study

**DOI:** 10.1007/s11845-026-04478-w

**Published:** 2026-06-16

**Authors:** Ebru Turan, Arzu Yüksel, Emel Bahadır Yılmaz

**Affiliations:** 1 Faculty of Medicine, Aksaray University, Aksaray, Türkiye; 2 Faculty of Health Sciences, Aksaray University, Aksaray, Türkiye; 3 Faculty of Health Sciences, Giresun University, Giresun, Türkiye

**Keywords:** Adolescents, Major depressive disorder, Problematic internet use, Sleep quality

## Abstract

**Background:**

Sleep disturbances and problematic internet use are important public health issues that may be more pronounced in individuals with major depressive disorder.

**Aim:**

This study aims to investigate sleep disturbance and problematic internet use levels in adolescents with and without major depressive disorder.

**Methods:**

This study was conducted with 177 adolescents who were admitted to a university training and research hospital between July 1, 2024 and January 30, 2025. The data collection was conducted using the “Adolescent Information Form,” the “Problematic Internet Use Scale-Adolescent Form,” the “DSM-5 Level 2 Sleep Disturbance Scale,” and the “Revised Child Anxiety and Depression Scale-Child Form.” Data were analyzed using Spearman correlation analysis and Multiple regression analysis.

**Results:**

The case group exhibited significantly higher mean scores than the control group on problematic internet use and sleep disturbances (*p* < 0.001). The predictors of problematic internet use were sleep disturbance (β = 0.237) and weekly internet usage time (β = 0.148, *p* < 0.05). The predictors of sleep disturbance in adolescents were child anxiety and depression (β = 5.556), total anxiety (β = 5.044), needing something specific to fall asleep (β = 3.182), and problematic internet use (β = 2.597, *p* < 0.05).

**Conclusions:**

Findings indicate a strong and bidirectional relationship between problematic internet use, sleep disturbances, and depressive symptoms in adolescents. Health professionals should routinely screen adolescents for problematic internet use, sleep disturbances, anxiety, and depression, and provide education on healthy digital habits and sleep hygiene.

## Introduction

 According to the World Health Organization (WHO), one in seven adolescents aged 10 to 19 experiences a mental disorder, with depression, anxiety, and behavioral disorders ranking among the leading causes of illness and disability in this age group [1]. Increasing rates of depression in adolescents have been associated with heightened internet addiction [2]. Furthermore, WHO reports indicate that between 2018 and 2022, problematic social media use among adolescents has increased, and approximately 12% of adolescents are at risk of problematic gaming. These findings have raised urgent concerns regarding the impact of digital technology on the mental health and overall well-being of young people [3].

In Turkey, as in many other countries, rates of depression and problematic internet usage among adolescents are steadily climbing. A study conducted during the pandemic in Turkey reported that approximately half of 3,058 adolescents experienced depression and anxiety symptoms. The findings indicated that girls, high school students, adolescents with parents of low educational levels, those with separated parents, increased social media use, and siblings numbering more than three were more likely to experience depression and anxiety [4]. Another study involving 2,780 adolescents in Istanbul found that 31.2% exhibited elevated depressive symptoms, with girls, those facing greater difficulties, lower psychological resilience, and lower perceived academic achievement identified as high-risk groups for depression [5]. Similarly, a study conducted with 1,081 adolescents in İzmir revealed a high prevalence of depressive symptoms, which were strongly associated with significant sleep problems [6]. The prevalence of depression and anxiety among Turkish adolescents, especially during the pandemic period, has markedly increased and is closely linked to factors such as psychological resilience, family structure, and social media usage.

Problematic internet use is also a significant issue among adolescents in Turkey. A study involving 1,558 adolescents reported that 21.1% exhibited problematic internet use, which was associated with low self-esteem as well as mental and physical health issues [7]. Another study conducted with 1,412 adolescents in Istanbul found this rate to be 18.5%, highlighting internet usage experience as a risk factor [8]. Adolescents who use the internet describe it using terms such as habit, friend, entertainment, basic necessity, and real life. They also report using the internet primarily for gaming purposes and experience related problems such as sleep disturbances and fatigue [9]. These data suggest that adolescents in Turkey engage heavily and multidimensionally with the internet; however, a considerable portion of this usage reaches problematic levels, negatively impacting both their psychological and physical health.

Problematic internet use has been identified as one of the factors associated with increasing depressive symptoms in adolescents [10]. One study reported that problematic internet use is a significant predictor of depressive symptoms in this population [11]. Another study found that the amount of time adolescents spend on passive browsing and social comparison online predicts higher depression scores [12]. A meta-analysis further demonstrated that problematic internet use is not only linked to depression but also associated with anxiety, loneliness, and other mental health outcomes, with problematic use increasing as subjective well-being decreases [13]. These findings suggest that, considering the emotional vulnerability characteristic of adolescence, excessive presence in digital environments may adversely affect psychological well-being.

Adolescents with high levels of problematic internet use have been reported to experience poorer sleep quality, insomnia, reduced sleep efficiency, and shorter sleep duration [14]. One study highlighted a relationship between problematic internet use and sleep quality among adolescents diagnosed with depression [15]. Based on these findings, the present study aims to investigate problematic internet use and sleep disturbance levels in adolescents with and without a diagnosis of Major Depressive Disorder (MDD).

Research questions:

1. Is there a difference in problematic internet use between adolescents with and without MDD?
2. Is there a difference in sleep disturbance between adolescents with and without MDD?
3. Is there a correlation between anxiety and depression, problematic internet use, and sleep disturbances in adolescents?

## Methods

### Study design

This study employed a cross-sectional and correlational design to determine the sleep disturbances and problematic internet use in adolescents with and without MDD. Descriptive cross-sectional studies are a commonly used and appropriate method for revealing the frequency and distribution of health indicators such as mental symptoms, problematic internet use, and sleep disturbances in a specific population at a particular time [16].

### Setting and participants

This study conducted between July 1, 2024 and January 30, 2025, in the Child and Adolescent Psychiatry Polyclinic of a university training and research hospital. The case group consisted of 85 adolescents who were newly diagnosed with depression using the Schedule for Affective Disorders and Schizophrenia for School-Age Children-Present and Lifetime Version, DSM-5 November 2016-Turkish Adaptation [17]. Inclusion criteria for the case group were: being newly diagnosed with MDD, being aged between 12 and 17 years, having no diagnosis other than depression, and not using any psychiatric medication. Exclusion criteria included lack of consent from the family for participation and unwillingness to participate in the study.

The control group consisted of 92 adolescents who were voluntarily willing to participate in the study and were assessed with the K-SADS-PL-DSM-5-T, with no psychiatric disorder diagnosis established. These adolescents attended the child and adolescent psychiatry outpatient clinic for relatively mild reasons such as routine developmental or health evaluations, brief counseling regarding academic stress, peer relationship difficulties, or parental concerns about typical adolescent behaviors, rather than for severe or persistent psychopathology. None of them had any prior history of psychiatric illnesses or long-term medical conditions according to clinical records and parental report. Exclusion criteria for the control group included meeting criteria for any current or past psychiatric disorder, presence of chronic medical or neurological illness, lack of family consent for participation, and unwillingness to participate in the study.

Power analysis performed with G*Power indicated that a sample of 118 adolescents was necessary for the study, given an effect size of 0.52, statistical power set at 80%, and a significance level of 5%. The study was carried out with a total of 177 adolescents, including 85 in the case group and 92 in the control group.

### Data collection

Data were collected face-to-face by a child and adolescent psychiatrist in the Child and Adolescent Psychiatry Polyclinic between July 1, 2024 and January 30, 2025. The data collection process took approximately 30 to 35 min.

### Measures

The data collection tools included the Adolescent Information Form, the Problematic Internet Use Scale-Adolescent Form (PIUS-A), the DSM-5 Level 2 Sleep Disturbance Scale (DSM-5-L2SDS), and the Revised Child Anxiety and Depression Scale-Child Form (RCADS-C).

### Adolescent information form

This form, developed by the researchers, includes questions about adolescents’ age, educational level, information about their parents, and the duration of their internet use [10, 11, 13].

### Problematic internet use scale - adolescent form (PIUS-A)

The scale was developed in Turkey by Ceyhan and Ceyhan and consists of 27 items [18]. The total score of the scale ranges from 27 to 135, with higher scores indicating increased problematic internet use among adolescents. The PIUS-A comprises three subdimensions: negative consequences of internet use, excessive use, and social benefit/social comfort. In the present study, the total Cronbach’s alpha coefficient was 0.94, with subscale coefficients of 0.87 for negative consequences, 0.73 for excessive use, and 0.79 for social benefit/social comfort.

### DSM-5 level 2 sleep disturbance scale - child form for ages 11–17

The Turkish validity and reliability study of the scale was conducted by Ozek-Erkuran et al. [19]. The scale consists of a total of eight items. This scale employs a five-point Likert rating format with possible total scores between 8 and 40. Higher scores indicate more severe symptoms associated with sleep disturbance. In the present study, the scale’s Cronbach’s alpha coefficient was also found to be 0.91.

### Revised child anxiety and depression scale - child form (RCADS-C)

It was developed to measure symptoms of depression and anxiety in children and adolescents aged 8–17 years according to DSM-IV criteria [20]. The Turkish validity and reliability study was conducted by Görmez et al. [21]. The scale consists of 47 items and includes six subscales: separation anxiety, social phobia, obsessive-compulsive disorder, panic disorder, generalized anxiety disorder, and major depressive disorder. In this study, the Cronbach’s alpha coefficient of the scale was 0.96. Subscale Cronbach’s alpha coefficients were as follows: separation anxiety 0.65, social phobia 0.87, obsessive-compulsive disorder 0.78, panic disorder 0.90, generalized anxiety disorder 0.80, and major depressive disorder 0.91.

### Statistical analyses

Data were analyzed using the Statistical Package for Social Sciences (SPSS) version 24.0 software. Descriptive statistics such as percentages, means, and standard deviations were used to analyze sociodemographic characteristics. Pearson Chi-Square and Fisher’s Exact Test were applied to compare descriptive characteristics between groups. The Kolmogorov-Smirnov test was employed to assess the normality of the data distribution. Since the data were not normally distributed, the Mann-Whitney U test was used to compare groups with and without a depression diagnosis, and Spearman correlation analysis was utilized to examine relationships between variables. Multiple regression analysis was conducted to identify predictors of sleep disturbance and problematic internet use in adolescents. A significance level of *p* < 0.05 was accepted for all statistical tests.

### Ethical consideration

Ethical approval was obtained from the University Ethics Committee (Decision No: 2024/50, dated 27.06.2024). Adolescents and their families who participated in the study were informed about the research, confidentiality of personal information was assured, and informed consent was obtained. The study was conducted in accordance with the principles of the Declaration of Helsinki throughout its duration.

## Results

### Sociodemographic characteristics

As shown in Table 1, the mean age of adolescents in the case group was 15.28 ± 1.57, while in the control group it was 14.91 ± 1.70. The proportion of female adolescents in the case group (75.3%) was higher than that in the control group (56.5%), and this difference was statistically significant (*p* = 0.009). No significant differences were found between the two groups in terms of age, number of siblings, birth order, grade level, parental education, or perceived family income (*p* > 0.05).

**Table 1:** Socio-demographic characteristics

	Case (*n* = 85)	Control (*n* = 92)		
Variables	*n* (%)	*n* (%)	χ²	*p*
Age (Mean ± SD)	15.28 ± 1.57	14.91 ± 1.70		
12–13 years	16 (18.8)	24 (26.1)	1.934	0.380
14–15 years	23 (27.1)	27 (29.3)		
16–17 years	46 (54.1)	41 (44.6)		
Gender				
Female	64 (75.3)	52 (56.5)	6.893	0.009
Male	21 (24.7)	40 (43.5)		
Number of siblings				
Only child	7 (8.2)	10 (10.9)	4.459	0.216
Two siblings	27 (31.8)	18 (19.6)		
Three siblings	34 (40.0)	37 (40.2)		
Four and more	17 (20.0)	27 (29.3)		
Birth order				
First	35 (41.1)	35 (38.0)	2.304	0.512
Second	31 (36.5)	28 (30.4)		
Third	14 (16.5)	19 (20.7)		
Fourth and more	5 (5.9)	10 (10.9)		
School grade				
6–7–8th grade	19 (22.4)	28 (30.4)	1.480	0.224
9–10–11–12th grade	66 (77.6)	64 (69.6)		
Mother’s education				
Primary school	57 (67.1)	60 (65.2)	0.097	0.952
High school	16 (18.8)	19 (20.7)		
University	12 (14.1)	13 (14.1)		
Father’s education				
Primary school	41 (48.3)	41 (44.6)	2.246	0.325
High school	32 (37.6)	30 (32.6)		
University	12 (14.1)	21 (22.8)		
Perceived family income level				
Low	8 (9.4)	8 (8.7)	0.661	0.770
Medium	71 (83.5)	80 (87.0)		
High	6 (7.1)	4 (4.3)		

### Internet usage characteristics

Table 2 compares the internet usage characteristics of adolescents in the case and control groups. There was a statistically significant difference in internet usage duration between adolescents in the case group (mean ± SD = 35.85 ± 30.45 h) and those in the control group (mean ± SD = 19.53 ± 15.54 h) (*p* = 0.008). A significantly greater proportion of adolescents with MDD (98.8%) possessed a mobile phone and accessed the internet through it, compared to those without MDD (88.0%) (*p* = 0.004). The duration of internet use via phone was also significantly longer in the case group (mean ± SD = 30.94 ± 29.35 h) than in the control group (mean ± SD = 14.74 ± 13.39 h) (*p* = 0.030). The most preferred device for internet access was the mobile phone in both groups and this difference was statistically significant (*p* = 0.015). A statistically significant difference was found between the two groups regarding parents’ marital status, with a higher number of divorced parents in adolescents with MDD (*p* = 0.010). The need for special objects while sleeping was greater in the case group compared to the control group (*p* = 0.031). No statistically significant differences were found between the two groups regarding owning a personal computer, having a personal room, or sharing the bedroom with others (*p* > 0.05).

**Table 2:** Internet use characteristics

	Case (*n* = 85)	Control (*n* = 92)		
Variables	*n* (%)	*n* (%)	χ²	*p*
Having a personal computer				
Yes	29 (34.1)	42 (45.7)	2.447	0.118
No	56 (65.9)	50 (54.3)		
Weekly internet usage time (Mean ± SD)*	35.85 ± 30.45	19.53 ± 15.54		
< 5 h/week	5 (5.9)	11 (12.0)	11.712	0.008
5–14 h/week	16 (18.8)	32 (34.8)		
15–39 h/week	38 (44.7)	36 (39.1)		
≥ 40 h/week	26 (30.6)	13 (14.1)		
Having a mobile phone				
Yes	84 (98.8)	81 (88.0)	8.124	0.004
No	1 (1.2)	11 (12.0)		
Accessing the internet via mobile phone				
Yes	84 (98.8)	81 (88.0)	8.124	0.004
No	1 (1.2)	11 (12.0)		
Weekly mobile phone internet usage time (Mean ± SD)*	30.94 ± 29.35	14.74 ± 13.39		
< 5 h/week	8 (9.5)	12 (14.8)	8.932	0.030
5–14 h/week	21 (25.0)	30 (37.0)		
15–39 h/week	33 (39.3)	31 (38.3)		
≥ 40 h/week	22 (26.2)	8 (9.9)		
Most preferred device for internet access				
Mobile phone	79 (92.9)	74 (80.4)	5.896	0.015
Computer	6 (7.1)	18 (19.6)		
Having a personal room				
Yes	60 (70.6)	74 (80.4)	2.329	0.127
No	25 (29.4)	18 (19.6)		
Parents’ marital status				
Married	67 (78.8)	85 (92.4)	6.705	0.010
Divorced/Separated	18 (21.2)	7 (7.6)		
Sharing a bedroom with others				
Alone	42 (49.4)	45 (48.9)	0.027	0.987
With mother	5 (5.9)	5 (5.4)		
With sibling(s)	38 (44.7)	42 (45.7)		
Needing something specific to sleep				
No	56 (65.9)	76 (82.6)	8.815	0.031
Light	11 (12.9)	10 (10.9)		
Sound	12 (14.1)	4 (4.3)		
Presence of another person	6 (7.1)	2 (2.2)		

* Usage time was self-reported

### Problematic internet use and sleep disturbance levels

Table 3 presents the comparison of mean scores for problematic internet use, sleep disturbance, anxiety, and depression scales between the groups. The mean total PIUS-A score was 66.89 ± 18.86 in the case group and 56.65 ± 16.72 in the control group, with the difference being statistically significant (*p* < 0.001). The mean total DSM-5-L2SDS score was 26.20 ± 7.67 in the case group and 17.47 ± 7.34 in the control group, and this difference was also statistically significant (*p* < 0.001).

**Table 3:** Comparison of problematic ınternet use, sleep disturbance, anxiety, and depression scale mean scores

Scales	Case				Control					
	Mean ± SD	Median	Min	Max	Mean ± SD	Median	Min	Max	Test (z)	*p*
Negative Consequences of the Internet	29.88 ± 10.92	27	14	62	25.65 ± 10.01	23	14	62	−2.869	0.004
Excessive Use	20.24 ± 5.34	20	6	29	18.21 ± 4.56	17	6	30	−2.544	0.011
Social Benefit/Social Comfort	16.76 ± 6.91	17	7	35	12.78 ± 5.07	12	7	29	−4.008	< 0.001
Total PIUS-A	66.89 ± 18.86	63	32	120	56.65 ± 16.72	53	31	112	−3.759	< 0.001
Total DSM-5–SDSS	26.20 ± 7.67	26	8	40	17.47 ± 7.34	16.5	8	40	−6.976	< 0.001

*z * Mann–Whitney U test, *PIUS-A * Problematic Internet Use Scale–Adolescent Form, *DSM-5–SDSS * DSM-5 Level 2 Sleep Disturbance Scale, *RCADS-CV * Revised Child Anxiety and Depression Scale–Child Version

### Correlation findings

Tables 4 presents the correlations among the scales. There is a moderate positive correlation between adolescents’ total PIUS-A and total DSM-5-L2SDS mean scores (*r* = 0.384, *p* < 0.001). A moderate positive correlation was also found between adolescents’ total RCADS-C and total PIUS-A mean scores (*r* = 0.451, *p* < 0.001). Additionally, a moderate positive correlation exists between adolescents’ total RCADS-C and total DSM-5-L2SDS mean scores (*r* = 0.515, *p* < 0.001). Table 5 presents of problematic internet use and sleep disturbance in adolescents. The variables explained 30% ofthe variance in problematic internet use (p 0.01). The most important predictors of problematic internet use weresleep disturbance (β = 0.237) and weekly internet usage time (β = 0.148, p 0.05). The variables explained 53% ofthe variance in sleep disorders (p 0.01). The most important predictors of sleep disturbance in adolescents were,respectively, total RCADS-CV (β = 5.556), total anxiety (β = 5.044), needing something specific to fall asleep (β =3.182), and total PIUS-A (β = 2.597, p 0.05).

**Table 4:** Correlation between problematic internet use, sleep disorders, and anxiety and depression scale scores in adolescents

		1	2	3	4	5	6
1. Negative Consequences of the Internet	*r*	1.000					
	*p*	-					
2. Excessive Use	r	0.652	1.000				
	p	< 0.001	-				
3. Social Benefit/Social Comfort	r	0.521	0.255	1.000			
	p	< 0.001	< 0.001	-			
4. Total PIUS-A	r	0.934	0.725	0.716	1.000		
	p	< 0.001	< 0.001	< 0.001	-		
5. Total DSM-5–SDSS	r	0.309	0.293	0.369	0.384	1.000	
	p	< 0.001	< 0.001	< 0.001	< 0.001	-	
6. Total RCADS-CV	r	0.411	0.278	0.431	0.451	0.515	1.000
	p	< 0.001	< 0.001	< 0.001	< 0.001	< 0.001	-

*PIUS-A * Problematic Internet Use Scale–Adolescent Form, *DSM-5–SDSS * DSM-5 Level 2 Sleep Disturbance Scale, *RCADS-CV * Revised Child Anxiety and Depression Scale–Child Version

Table 5 presents of problematic internet use and sleep disturbance in adolescents. The variables explained 30% of the variance in problematic internet use (p 0.01). The most important predictors of problematic internet use were sleep disturbance (β = 0.237) and weekly internet usage time (β = 0.148, p 0.05). The variables explained 53% of the variance in sleep disorders (p 0.01). The most important predictors of sleep disturbance in adolescents were, respectively, total RCADS-CV (β = 5.556), total anxiety (β = 5.044), needing something specific to fall asleep (β =3.182), and total PIUS-A (β = 2.597, p 0.05).

**Table 5:** Predictors of problematic internet use and sleep disturbance in adolescents

Variables	B	SE	β	t	*p*
Total PIUS-A (*R* = 0.548, R^2^ = 0.301, F = 7.983, *p* < 0.01)					
Total DSM-5–SDSS	0.504	0.191	0.237	2.642	0.009
Total anxiety	−0.035	0.330	−0.043	−0.106	0.916
Total RCADS-CV	0.286	0.279	0.453	1.023	0.308
Group	3.173	3.257	0.086	0.974	0.331
Age	−1.551	0.784	−0.139	−1.977	0.050
Gender	0.438	2.670	0.011	0.164	0.870
Having a personal computer	−4.040	2.588	−0.108	−1.561	0.120
Weekly internet usage time	0.109	0.053	0.148	2.067	0.040
Parents’ marital status	−5.868	3.565	−0.111	−1.646	0.102
Total DSM-5–SDSS (*R* = 0.727, R^2^ = 0.528, F = 16.783, *p* < 0.01)					
Total PIUS-A	0.076	0.029	0.163	2.597	0.010
Total anxiety	−0.606	0.120	−1.596	−5.044	< 0.001
Total RCADS-CV	0.556	0.100	1.878	5.556	< 0.001
Group	−1.772	1.260	−0.102	−1.406	0.162
Age	0.326	0.309	0.062	1.057	0.292
Gender	−0.316	1.044	−0.017	−0.302	0.763
Having a personal computer	1.430	1.052	0.081	1.359	0.176
Weekly internet usage time	0.021	0.021	0.061	1.010	0.314
Parents’ marital status	2.476	1.388	0.100	1.783	0.076
Having a private room at home	0.021	1.147	0.001	0.018	0.985
Needing something specific to fall asleep	1.883	0.592	0.182	3.182	0.002

*PIUS-A* Problematic Internet Use Scale–Adolescent Form, *DSM-5–SDSS * DSM-5 Level 2 Sleep Disturbance Scale, *RCADS-CV * Revised Child Anxiety and Depression Scale–Child Version

### Regression findings

The variables explained 30% of the variance in problematic internet use (*p* < 0.01). The most important predictors of problematic internet use were sleep disturbance (β = 0.237) and weekly internet usage time (β = 0.148, *p* < 0.05). The variables explained 53% of the variance in sleep disorders (*p* < 0.01). The most important predictors of sleep disturbance in adolescents were, respectively, total RCADS-CV (β = 5.556), total anxiety (β = 5.044), needing something specific to fall asleep (β = 3.182), and total PIUS-A (β = 2.597, *p* < 0.05).

## Discussion

The purpose of this research was to compare the problematic internet use and sleep disturbance levels of adolescents with and without MDD. Adolescents with MDD demonstrated longer internet usage time compared to their non-diagnosed peers. Additionally, one of the two most important predictors of problematic internet use is weekly internet usage time. Adolescents using the internet 6–8 h daily had significantly higher odds of depressive symptoms than peers with limited or no use [22]. Similarly, higher internet usage was associated with increased depressive symptoms over time, particularly among girls, who tend to engage more in social and interactive online activities than boys [23]. Beyond duration and content, several mediating factors appear to play a key role in the relationship between internet use and depression. Previous studies have shown that problematic or excessive internet use can disrupt family relationships, lead to conflicts and reduced communication with parents, impair academic performance through reduced concentration and study time, and adversely affect sleep patterns by delaying bedtime and shortening total sleep duration [24, 25]. In addition, studies show that as internet gaming disorder increases, the risk of depression also rises, and that gaming disorder is associated with depression, particularly through impaired sleep quality [26, 27]. Furthermore, internet use time negatively impacts mental health, manifesting as shorter sleep duration and decreased parent-adolescent communication [28]. Overall, these findings highlight the complex and multifaceted relationship between different types of internet use and mental health outcomes, emphasizing the need for targeted interventions that consider both risk and protective factors.

In this study, adolescents diagnosed with MDD exhibited significantly greater sleep disturbance compared to adolescents without this diagnosis. Additionally, childhood anxiety and depression are among the most important predictors of sleep disturbance. Several clinical assessments confirm that adolescents with MDD have reduced total sleep time and impaired sleep quality compared to controls, supporting sleep disturbance as a characteristic feature of adolescent depression [29]. Adolescents with MDD frequently experience significantly greater sleep disturbances. The severity of depressive symptoms correlates positively with sleep disturbance scores, suggesting a close link between poor sleep and depression severity [30]. Adolescents with depression exhibit stronger correlations between depressive symptoms and sleep problems compared to younger children. Sleep difficulties include overtiredness, trouble sleeping, and irregular sleep duration [31]. Objective sleep measures in late adolescence indicate that short sleep duration predicts subsequent major depressive episodes in early adulthood, independent of prior depression [32]. Factors such as irregular sleep patterns, social media use, and pre-sleep cognitive arousal contribute to sleep disturbances in depressed adolescents [33]. In sum, sleep disturbances in adolescents with MDD are both a hallmark symptom and a potential risk factor for the persistence and worsening of depression.

One of the predictors of sleep disturbance in adolescents was problematic internet usage. Recent studies have consistently demonstrated a significant association between problematic internet use and increased sleep problems as well as reduced sleep quality among adolescents, independent of depression status [34, 35]. Cross-sectional findings among adolescents aged 13–15 indicate a strong relationship between internet addiction and sleep disorders, with a substantial proportion of affected individuals also experiencing sleep disturbances [36]. In addition, late-night internet use, particularly after 9 PM, has been associated with poorer sleep quality, shorter total sleep duration, delayed bedtimes, and increased internalizing symptoms such as depression and anxiety [37]. Supporting these findings, large-scale clinical evidence shows that problematic internet use is linked to a range of sleep-related difficulties, including insomnia, excessive daytime sleepiness, and disrupted sleep–wake patterns in both depressed and non-depressed adolescents [38]. Overall, these results highlight the importance of addressing problematic internet use in adolescence as a key factor in promoting healthy sleep patterns and supporting mental well-being.

### Limitations

There are some limitations. Because the research was conducted at only one center, the findings may not be widely applicable to larger or different populations. Self-report measurement tools were used, and the collected data are limited to the constructs measured by these scales. Regression studies should be conducted to identify predictors of problematic internet use and sleep problems in adolescents with MDD. The cross-sectional design of this study does not allow for causal inferences regarding the directionality of the relationships between problematic internet use, sleep disturbances, anxiety, and depressive symptoms. Longitudinal designs are needed to clarify temporal and potentially bidirectional effects. Although several clinically relevant variables were examined, potential confounding factors such as medication use, comorbid psychiatric diagnoses, substance use, and family socioeconomic status were not comprehensively controlled for, which may have influenced the observed associations. Finally, it should be acknowledged that the control group was recruited from adolescents attending a child and adolescent psychiatry outpatient clinic, rather than from a community or school-based sample. Although these participants did not meet diagnostic criteria for any psychiatric disorder and were largely referred for routine evaluations or brief counseling about normative developmental or academic issues, they may not fully represent a general, non-clinical adolescent population. This sampling approach may therefore limit the generalisability of our findings, particularly with respect to the comparison between adolescents with MDD and their “healthy” peers, and future studies should include community-based control groups to better capture the full spectrum of adolescent mental health.

## Conclusion

In summary, the findings indicate a strong and bidirectional relationship between problematic internet use, sleep disturbances, and depressive symptoms in adolescents. Adolescents with MDD show higher internet use and more severe sleep problems, while weekly internet use time emerges as an important predictor of both problematic internet use and sleep disturbance. Excessive and late-night internet use appears to worsen sleep quality and is linked with increased depressive symptoms, suggesting a reinforcing cycle between digital behavior and mental health. In addition, needing specific conditions to fall asleep reflects poor sleep hygiene and is associated with delayed sleep onset and reduced sleep quality. Together, these results suggest that both behavioral (internet use patterns) and sleep-related habits play a key role in adolescent mental health, highlighting the importance of early preventive and targeted interventions. Based on these findings, preventive interventions should focus on limiting excessive and late-night internet use among adolescents. Sleep hygiene education programs may be useful in promoting more regular and independent sleep routines. Family-based interventions that encourage healthier communication patterns and reduce conflict related to internet use may also be beneficial. In addition, screening for sleep-onset dependencies and problematic internet use in school and clinical settings could help identify at-risk adolescents early and support timely intervention.
